# Parental care influences leukocyte telomere length with gender specificity in parents and offsprings

**DOI:** 10.1186/s12888-014-0277-9

**Published:** 2014-10-03

**Authors:** Masanori Enokido, Akihito Suzuki, Ryoichi Sadahiro, Yoshihiko Matsumoto, Fumikazu Kuwahata, Nana Takahashi, Kaoru Goto, Koichi Otani

**Affiliations:** Department of Psychiatry, Yamagata University School of Medicine, 2-2-2 Iidanishi, Yamagata, 990-9585 Japan; Department of Anatomy and Cell Biology, Yamagata University School of Medicine, 2-2-2 Iidanishi, Yamagata, 990-9585 Japan

**Keywords:** Care, Gender specificity, Healthy subjects, Parental rearing, PBI, Telomere length

## Abstract

**Background:**

There have been several reports suggesting that adverse childhood experiences such as physical maltreatment and long institutionalization influence telomere length. However, there has been no study examining the relationship of telomere length with variations in parental rearing. In the present study, we examined the relationship of leukocyte telomere length with parental rearing in healthy subjects.

**Methods:**

The subjects were 581 unrelated healthy Japanese subjects. Perceived parental rearing was assessed by the Parental Bonding Instrument consisting of the care and protection factors. Leukocyte relative telomere length was determined by a quantitative real-time PCR method for a ratio of telomere/single copy gene.

**Results:**

In the multiple regression analyses, shorter telomere length in males was related to lower scores of paternal care (*β* = 0.139, *p* < 0.05), while that in females was related to lower scores of maternal care (*β* = 0.195, *p* < 0.01).

**Conclusion:**

The present study suggests that there is linear relationship between parental care and telomere length which covers both lower and higher ends of parental care, and that the effects of parental care on telomere length are gender-specific in parents and offsprings.

## Background

Telomeres are repetitive DNA sequences (TTAGGG)n located at the ends of chromosomes and play a crucial role in preventing chromosome fusion and in maintaining genome stability [1,2]. Telomere length is maintained by a cellular enzyme, telomerase, in germ cells and stem cells, while most somatic cells have very low telomerase activity, thus leading to telomere length shortening with cellular division [1,2]. When telomere length reaches a critical point, cellular senescence is triggered, cell division ceases, and the cell dies [1,2]. Thus, telomere length represents a biological marker for cellular aging. Prospective studies have shown that shorter telomere length is a predictor of coronary heart disease [3], cancer [4], progression of diabetic nephropathy in patients with type 1 diabetes [5], dementia in post-stroke patients [6], and mortality [7]. Shorter telomere length has also been associated with mood disorders, schizophrenia, mild cognitive impairment, and Alzheimer disease [8].

It has been reported that telomere length is influenced by a wide range of factors such as age, gender, race, smoking, physical activity, socioeconomic status, obesity, multivitamin intake, alcohol consumption, and hormone replacement therapy [2], although findings are inconsistent [9]. In relation to psychological factors, shorter leukocyte telomere length is associated with current life stress [10] and personality traits such as pessimism [11] and hostility [12]. Recent studies have focused on the association between telomere length and early-life environment, and these studies have suggested that adverse experiences such as physical abuse and long institutionalization accelerate telomere length shortening [8,13,14], with inconsistent findings [15,16]. However, there has been few study examining the relationship of telomere length with variations in parental rearing which has a continuum ranging from a positive pole to a negative pole [17].

In attachment theory, Bowlby [18] has proposed that the crucial roles of parents are, first, to provide a secure base and, second, to encourage a child to explore from that base, developing secure attachment in the child. The child with secure attachment grows up to be secure, self-reliant, and co-operative. In contrast, parents who are unresponsive to a child’s desire for care and do not allow the child’s progressive independence create anxious attachment in the child. Strongly influenced by attachment theory [18], Parker et al. [19] developed the Parental Bonding Instrument (PBI), which assesses perceived rearing attitudes of subject’s parents during the first 16 years. The PBI contains the care factor and the protection factor. In the care factor, high scores suggest affection, emotional warmth, and empathy, while low scores indicate emotional coldness, indifference, and neglect. In the protection factor, high scores suggest control, overprotection, and intrusion, while low scores indicate allowance of independence and autonomy. The PBI has been widely used in the field of developmental psychiatry, e.g., studies on the effects of parental rearing on vulnerability factors to depression [20-22]. Meanwhile, low care and high protection assessed by the PBI are shown to be associated with physical diseases such as coronary heart disease [23], gastroesophageal reflux disease [24], Crohn’s disease [25], and inflammatory bowel disease [26].

These discussions lead to the hypothesis that telomere length is influenced by variations in parental rearing during childhood in both positive and negative manners. Therefore, we studied the effects of parental rearing assessed by the PBI on leukocyte telomere length in healthy subjects.

## Methods

The subjects were 581 mentally and physically healthy Japanese subjects who were recruited from medical students and hospital staffs living in Yamagata Prefecture. Data collection was performed from September 2003 to October 2011. Absence of a current or past history of psychiatric disorders were confirmed by interviews by well-trained psychiatrists and a questionnaire on psychiatric treatment and diagnosis. Six items selected from the Structured Clinical Interview for DSM-IV Axis I Disorders [27] were used for the psychiatric interview. They were A1 for major depressive episode, A16 for manic episode, B1 for delusions, B6 for hallucinations, E2 for alcohol abuse, and F68 for anxiety disorders. All subjects declared in the check sheet that they had no serious physical diseases. None had his or her parents divorced or deceased before the age of 16. Demographic characteristics of the subjects were shown in Table 1. Three hundred forty subjects were males, and 241 were females. The mean ± SD (range) of age was 23.4 ± 1.7 (20–29) years. This study was conducted as part of a series of studies e.g., [20-22,28] on genetic and environmental factors involved in characterization of personality traits in healthy subjects. Two-hundred seven subjects (36%) out of the present sample overlapped with the subjects of our previous study which examined the relationship between telomere length and personality traits [28]. The study protocol was approved by the Ethics Committee of the Yamagata University School of Medicine. After complete description of the study, written informed consent was obtained from all subjects. The study was conducted according to the principles of the Helsinki Declaration.

**Table 1:** **Characteristics of subjects, relative telomere length, and PBI scores**

	**Males**	**Females**	***t***	***p***
Number of subjects (n)	340	241		
Age (years, mean ± SD)	23.4 ± 1.6	23.5 ± 1.9	0.603	0.547
Relative telomere length (z-score, mean ± SD)	−0.1 ± 1.0	0.1 ± 1.0	1.615	0.107
PBI (score, mean ± SD)				
Paternal care	23.8 ± 6.8	25.0 ± 6.6	2.232	0.026
Paternal protection	10.4 ± 5.5	10.9 ± 5.5	1.070	0.285
Maternal care	27.8 ± 5.2	29.0 ± 5.8	2.459	0.014
Maternal protection	11.7 ± 5.9	12.1 ± 6.1	0.662	0.508

PBI; Parental Bonding Instrument, SD; standard deviation.

The PBI is a self-report scale comprised of 25 items [19]. In the present study, parental rearing was assessed by the Japanese version of the PBI [29], which has been shown to have high reliability, i.e., high internal consistency and stability over time, and high content, concurrent, and construct validity [29].

Genomic DNA was extracted from peripheral leucocytes using a QIAamp DNA Blood Kit (Qiagen, Tokyo, Japan), and was stored at −80°C before PCR amplification. Leukocyte relative telomere length, assessed by a ratio of telomere/single copy gene with the mean data from the triplicate runs, was determined by a quantitative real-time PCR method of Cawthon [30] with the several modifications [28]. Relative telomere length was expressed as a standardized z-score.

Gender differences and inter-correlations in relative telomere length, PBI scores (paternal care, paternal protection, maternal care, and maternal protection), and age were tested by the Student’s t-test and the Pearson’s linear regression test, respectively. The effects of care and protection scores of parents on relative telomere length were analyzed by the stepwise multiple regression analysis where the dependent variable was relative telomere length, and the independent variables were the PBI scores and age. The analyses were performed in males and females separately, because previous studies showed gender specificity in the effects of parental rearing on various psychological factors, i.e., personality traits [20], interpersonal sensitivity [21], and cognitive vulnerability to depression [22]. All statistical analyses were performed by SPSS 14.0 J for Windows (SPSS Japan Inc, Tokyo, Japan), and a p value of less than 0.05 (two-tailed) was regarded as significant.

## Results

The relative telomere length and the PBI scores in males and females are shown in Table 1. Females had higher scores of paternal care (*p* = 0.026) and maternal care (*p* = 0.014) than males (Table 1).

**Figure 1:**
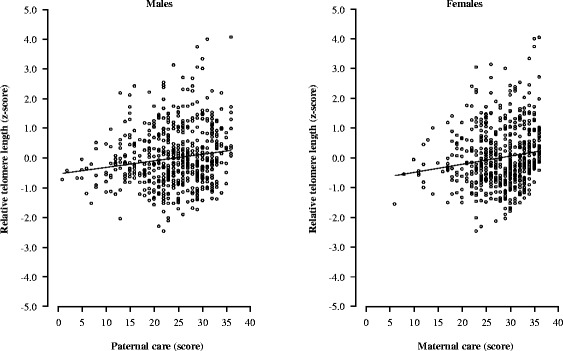
**Relationships of relative telomere length with paternal care in males (left) and maternal care in females (right).** Telomere length was expressed as a standardized z-score.

Table 2 shows the correlations among relative telomere length, PBI scores, and age. Tables 3 shows the results of the stepwise multiple regression analyses of relative telomere length with PBI scores and age in males and females. In males, shorter telomere length was related (*R*^*2*^ = 0.041, *p* = 0.001, Cohen’s *f* ^*2*^ = 0.043) to lower scores of paternal care (*p* = 0.010) and higher age (*p* = 0.005), while in females shorter telomere length was related (*R*^*2*^ = 0.038, *p* = 0.002, Cohen’s *f* ^*2*^ = 0.040) to lower scores of maternal care (*p* = 0.002) (Table 3 and Figure 1).

**Table 2:** **Correlations among relative telomere length, PBI scores, and age in males (above diagonal) and females (below diagonal)**

	**1**	**2**	**3**	**4**	**5**	**6**
1. Relative telomere length	-	0.136*	−0.057	0.099	−0.012	−0.149**
2. Paternal care	0.159*	-	−0.364***	0.471***	−0.187**	0.024
3. Paternal protection	0.005	−0.327***	-	−0.175**	0.459***	−0.028
4. Maternal care	0.195**	0.440***	−0.297***	-	−0.379***	−0.012
5. Maternal protection	−0.033	−0.175**	0.582***	−0.381***	-	−0.015
6. Age	−0.121	−0.055	0.101	−0.077	−0.002	-

PBI; Parental Bonding Instrument.

Figures on the Table show Pearson’s correlation coefficient.

*p < 0.05, **p < 0.01, ***p < 0.001.

**Table 3:** **Stepwise multiple regression analyses of relative telomere length with PBI scores and age in males and females**

	**Males**					**Females**				
	***β***	***t***	***SE***	***p***	***95% CI***	***β***	***t***	***SE***	***p***	***95% CI***
Paternal care	0.139	2.606	0.052	0.010	0.033, 0.237	-	-	-	-	-
Paternal protection	-	-	-	-	-	-	-	-	-	-
Maternal care	-	-	-	-	-	0.195	3.076	0.060	0.002	0.067, 0.304
Maternal protection	-	-	-	-	-	-	-	-	-	-
Age	−0.152	−2.854	0.032	0.005	−0.156, −0.029	-	-	-	-	-
Fit of model	*R* ^*2*^ = 0.041, *p* = 0.001					*R* ^*2*^ = 0.038, *p* = 0.002				

*95% CI* = 95% confidence interval, PBI; Parental Bonding Instrument, *SE* = standard error, “-”; not significant.

## Discussion

In the present study, there were positive correlations between parental care and leukocyte telomere length in both males and females, i.e., the subjects reporting their parents as emotionally cold, indifferent, and neglectful were more likely to have shorter telomere lengths. The present result is in line with the previous studies reporting that severe negative life-events during childhood were associated with shorter telomere length [8,13,14]. However, the present study is the first to show the linear relationship between parental care and telomere length which covers both lower and higher ends of parental care. The latter result suggests that higher parental care may have protective effects against telomere shortening in the face of other stressors which may influence telomere length, e.g., financial difficulties, bullying, and physical diseases [8].

The exact mechanism(s) explaining the positive correlation between parental care and telomere length remains unclear, but one possible mechanism may be an involvement of the hypothalamus-pituitary-adrenal (HPA) function. It was reported that mice reared under the condition of low maternal care or maternal separation displayed impaired negative feedback sensitivity to glucocorticoid, and consequently increased adrenocorticotropic hormone and corticosterone response to acute stress [31,32]. In human, subjects with low parental care assessed by the PBI exhibited increased corticotropin-releasing factor in cerebrospinal fluid [33], increased cortisol awaking response [34], and increased cortisol response to stress test [35]. Meanwhile, an in vitro study showed that exposure of human T lymphocytes to cortisol caused a reduction in telomerase activity [36]. In vivo, elevated urinary nocturnal cortisol levels [37] and increased cortisol response to stress test [38] were associated with shorter telomere length in healthy women. Taken together, it is possible that the relationship between decreased parental care and short telomere length observed in the present study is mediated by impaired HPA function.

Another finding of this study is that the effects of parental care on telomere length were gender-specific, i.e., telomere length in males was correlated with paternal care, while that in females was correlated with maternal care. Our previous studies showed that parental rearing by the same-gender parents had greater impacts on personality traits of harm avoidance and interpersonal sensitivity in healthy subjects [20,21]. On the other hands, these personality traits have been reported to be associated with overeating and smoking [39-42] which accelerate telomere shortening [2]. Therefore, the gender-specific effects of parental rearing on telomere length may be mediated by impaired personality development leading to the behaviors accelerating telomere shortening. However, to clarify the biological mechanisms involved in the gender specificity, further study is needed to examine the effects of parental rearing on biological markers including HPA function in males and females separately.

There are several limitations in the present study. Firstly, the present study was a cross-sectional design, i.e., the PBI and telomere length were assessed simultaneously. This raises the possibility that the parental rearing assessed in this study may be biased by perception of subjects. However, Parker [43] showed that the PBI scores provided by their subjects reflected the actual parenting assessed by significant others with acceptable validity. In addition, long-term stability of the PBI scores was shown in a 20-year follow-up study [44]. Therefore, this possibility seems unlikely. On the other hand, a causal relationship between the PBI and telomere length cannot be established in a cross-sectional design, necessitating a longitudinal study. Secondly, the present study did not assess other factors which may influence telomere length, e.g., smoking, excessive alcohol consumption, unhealthy eating, body mass index, childhood maltreatment, socioeconomic status, physical activity [2], and current life stress [10]. In particular, our subjects with low parental care might have received childhood maltreatment from their parents, and this may be driving the association between low parental care and short telomere length. Also, paternal age at birth might have confounded the present results [45], i.e., older father have higher chances of having higher incomes and hence displaying higher care. Therefore, in further studies on the relationship between telomere length and the PBI, the assessment of these factors is needed. Lastly, the subjects of this study were all Japanese, and they were all medical students or hospital staffs, i.e., well-educated people. Although this homogeneity of subjects may be an advantage in genetic association studies, the fact may make it difficult to extrapolate the present results to general populations or other ethnic groups.

## Conclusions

The present study suggests that there is linear relationship between parental care and telomere length which covers both lower and higher ends of parental care, and that the effects of parental care on telomere length are gender-specific in parents and offsprings.
